# Efficient Gene Transfer and Gene Editing in Sterlet (*Acipenser ruthenus*)

**DOI:** 10.3389/fgene.2018.00117

**Published:** 2018-04-06

**Authors:** Ji Chen, Wei Wang, Zhaohui Tian, Ying Dong, Tian Dong, Hua Zhu, Zuoyan Zhu, Hongxia Hu, Wei Hu

**Affiliations:** ^1^State Key Laboratory of Freshwater Ecology and Biotechnology, Institute of Hydrobiology, Chinese Academy of Sciences, Wuhan, China; ^2^Beijing Fisheries Research Institute, Beijing Key Laboratory of Fishery Biotechnology, Beijing, China; ^3^Qingdao National Laboratory for Marine Science and Technology, Qingdao, China

**Keywords:** sterlet, gene transfer, gene editing, EGFP, *no tail*

## Abstract

The sturgeon (Acipenseriformes) is an important farmed species because of its economical value. However, neither gene transfer nor gene editing techniques have been established in sturgeon for molecular breeding and gene functional study until now. In this study, we accomplished gene transfer and gene editing in sterlet (*Acipenser ruthenus*), which has the shortest sexual maturation period of sturgeons. The plasmid encoding enhanced green fluorescent protein (EGFP) was transferred into the embryos of sterlet at injection concentration of 100 ng/μL, under which condition high survival rate and gene transfer rate could be achieved. Subsequently, exogenous EGFP was efficiently disrupted by transcription activator-like effector nucleases (TALENs) or clustered regularly interspaced short palindromic repeats (CRISPR)/Cas9 nuclease/guide RNA (gRNA), with injection concentrations of 300 ng/μL TALENs, or 100 ng/μL Cas9 nuclease and 30 ng/μL gRNA, respectively, under which condition high survival rate and gene mutation rate could be achieved. Finally, the endogenous gene *no tail* in sterlet was successfully mutated by Cas9 nuclease/gRNA. We observed the CRISPR-induced *no tail* mutation, at a high efficiency with the mutant P0 embryos displaying the expected phenotype of bent spine and twisted tail.

## Introduction

Since the first batch of transgenic fish was produced, more than 35 fish species have been modified using exogenous genes, which included commercial aquaculture fish such as common carp (*Cyprinus carpio*), grass carp (*Ctenopharyngodon idellus*), Nile tilapia (*Oreochromis niloticus*), channel catfish (*Ietalurus punetaus*), rainbow trout (*Oncorhynchus mykiss*), coho salmon (*Oncorhynchus kisutch*), Atlantic salmon (*Salmo salar*), and mud loach (*Misgurnus mizolepis*) (Hu and Zhu, 2010), as well as laboratory model fish such as zebrafish (*Danio rerio*) and medaka (*Oryzias latipes*). Breeds with economically desirable characteristics combined by transgenic technique will have prospective applications in aquaculture and fish breeding (Gui and Zhu, 2012). The approval of the AquAdvantage Salmon^TM^ by Food and Drug Administration (FDA) of United States in 2015 was an encouraging case of the application of transgene breeding in aquaculture.

Targeted gene editing has rapidly developed in the last 10 years. Reverse genetics research on animals and plants was largely pushed ahead by zinc finger nucleases (ZFNs), transcription activator-like effector nucleases (TALENs) and clustered regularly interspaced short palindromic repeats (CRISPR) (Gaj et al., 2013), which showed great potential to modify genes of interest (Ding et al., 2013). Until now, targeted gene editing has been reported in zebrafish, medaka, Nile tilapia, rainbow trout, common carp, channel catfish, rohu (*Labeo rohita*), Chinese tongue sole (*Cynoglossus semilaevis*), rice field eel (*Monopterus albus*), and so on (Doyon et al., 2008; Ansai et al., 2014; Li et al., 2014; Yano et al., 2014; Chakrapani et al., 2016; Qin et al., 2016; Zhong et al., 2016; Cui et al., 2017; Feng et al., 2017).

The sturgeon (Acipenseriformes) is one of the oldest fish orders. They have important commercial values because of their delicious meat rich in various essential amino acids (such as lysine, leucine, isoleucine, methionine, threonine, tryptophan, phenylalanine, valine, and so on) and unsaturated fatty acids, and especially because they are the source of caviar. However, wild sturgeons are at endangered state due to overexploitation and destruction of their ecological environment (Billard and Lecointre, 2000). Genetic breeding of farmed sturgeon has become an important tool for conservation of wild sturgeon resources, as well as to meet the demand for human consumption of sturgeon products and food.

However, most sturgeon species have long sexual maturation periods, usually 6–12 years for males and 10–18 years for females in natural environments, which is a disadvantage in conventional genetic breeding. Thus, molecular breeding technologies such as gene transfer and targeted gene editing are desirable for sturgeon breeding.

In addition, sturgeons are between cartilaginous fish and teleostean, which attracts the interests of evolutionary scientists in terms of how their genes were conserved in polyploid genomes over the long history. Unfortunately, the study of gene function in sturgeon is limited to gene cloning and expression patterns (Abdolahnejad et al., 2015; Fajkowska et al., 2016; Song et al., 2016; Yarmohammadi et al., 2017), due to lacking of efficient gene manipulation technologies.

Among sturgeons, sterlet (*Acipenser ruthenus*) has the shortest sexual maturation period, usually 2–3 years for farmed males and 3–4 years for females, which shortens the time for genetic breeding cycle and the establishment of techniques to manipulate the genome. Meanwhile, the tetraploid sterlet is the sturgeon with lowest polyploidy genome, which makes it relatively easier to study the evolution and function of paralogous genes. Thus, sterlet could be regarded as an ideal model for sturgeons.

In recent years, a few laboratories tried to micro-manipulate embryos in sterlet. For example, one research team microinjected *dead end* morpholino into the embryos of sterlet. They observed the retardation of proliferation and migration of primordial germ cells, which was traced by FITC-dextrans, and obtained fertile fish (Linhartová et al., 2015; Saito and Psenicka, 2015). However, gene transfer and gene editing, the most common techniques in current fish molecular genetic breeding and gene functional study, have not yet been reported in any sturgeons including sterlet.

In this study, we established gene transfer and gene editing techniques in sterlet, which provided technical support for the validation of gene function, the study of traits with economical value, and the production of genetically modified sturgeon.

## Materials and Methods

### Ethics Statement

All animal experiments were conducted in accordance with the Guidelines and Protocols for the Care and Use of Laboratory Animals and were approved by the Institute of Hydrobiology, Chinese Academy of Sciences (Approval ID: keshuizhuan08529).

### Artificial Fertilization

Sexually mature sterlet was cultured in sturgeon fish farm of Beijing Fisheries Research Institute located in Fangshan district, Beijing. During spawning season, individuals with well-developed gonads were transferred to the laboratory. Mixture of luteinizing hormone releasing hormone analog (LHRHa, 10 μg per 1 kg fish body weight) and domperidone (DOM, 1 mg per 1 kg body weight) was injected once or twice, to induce spermiation or ovulation, respectively. The semen was diluted 80–100 times using 0.3× Danieau buffer before artificial fertilization.

### Preparation of Plasmids for Microinjection

The cytomegalovirus (CMV) promoter, the coding region for monomeric red fluorescent protein (mRFP) and SV40 sequence was cloned into the plasmid pEGFP-C1 (Clontech) to construct pGFP-RFP, which could express both green and red fluorescent proteins (Supplementary Figure 2A). The two plasmids were linearized by ClaI (NEB, R0197).

### Preparation of TALENs mRNA, Cas9 mRNA/gRNA, and Cas9 Nuclease/Guide RNA Targeting Exogenous EGFP

TALENs and guide RNA (gRNA) sequences were designed to target the core coding region of enhanced green fluorescent protein (EGFP), ACC TAC GGC (Barondeau et al., 2003). TALENs plasmids were constructed using the Golden Gate method (Cermak et al., 2011). The binding-site sequences were 5′-TGG CCC ACC CTC GTG ACC A-3′ for the left arm, and 5′-AGC GGC TGA AGC ACT GCA-3′ for the right arm (Supplementary Figure 2B). The FokI backbone plasmids were PCS2-FokI-KKR and PCS2-FokI-ELD (Liu et al., 2014). The reconstructed plasmids were linearized by NotI (NEB, R0189), then used as templates in an *in vitro* transcription system using a mMESSAGE mMACHINE SP6 kit (Ambion, AM1340). Finally, the resultant mRNA for the left and right arms were mixed in an equal proportion, and diluted to 100, 200, 300, 400, and 500 ng/μL (total mRNA).

The target site sequence of Cas9 on EGFP was GGT CAG GGT GGT CAC GAG GG (Supplementary Figure 2C). The gRNA was synthesized according to Hwang’s et al. (2013) method. A pair of forward (F) and reverse (R) oligos was synthesized using the sequences: F, 5′-TAG GTC AGG GTG GTC ACG AGG G-3′; R, 5′-AAA CCC CTC GTG ACC ACC CTG A-3′. Oligos were mixed at a final concentration of 10 mM each and heated at 95°C for 5 min, followed by a slow cooling to 37°C. Plasmid DR274 (Addgene plasmid 42250) was digested using BsaI (NEB, R0535) and ligated with the paired oligos. Then, the ligation mix was transformed into competent *E. coli* cells. Positive clones were confirmed by PCR using M13(-47) primer and oligo R. The reconstructed plasmid was digested by DraI (NEB, R0129), and a 285 bp DNA fragment was recycled and cleaned up. This fragment was then used as a template in an *in vitro* transcription system using a MEGAshortscript kit (Ambion, AM1354). The plasmid pCS2-nls-zCas9-nls (Addgene plasmid 47929) was linearized by NotI, and *in vitro* transcribed using a mMESSAGE mMACHINE SP6 kit. The Cas9 mRNA and gRNA was mixed in a proportion of 20:1 and the Cas9 mRNA was diluted to 100, 200, 300, 400, and 500 ng/μL. The Cas9 nuclease protein was purchased from Life Technologies (B25640), mixed with gRNA before injection in a proportion of 3:1, and then diluted to 100, 200, 300, 400, and 500 ng/μL.

### Preparation of Cas9 Nuclease/gRNA Targeting Endogenous *no tail* Gene

Three gRNAs were designed to target the first exon of the *no tail (ntl*) gene in sterlet. The target sites were gRNA1-GGC TTG AAG ACG TGG ATC TT, gRNA2-GGA GAG GGA TAT TAA AGT G, and gRNA3-GGA TCT TTG GAC GAA GTT TA (Supplementary Figure 2D). Each gRNA was transcribed and mixed with Cas9 nuclease.

### Microinjection

About 50 fertilized eggs of sterlet were spread onto each glass dish. For each embryo, 2 nL of prepared sample was microinjected into the animal pole of one-cell stage embryos using a glass capillary needle.

The linearized pEGFP-C1 plasmid was microinjected at 25, 50, 100, 150, or 200 ng/μL, with 0.1% phenol red used as a negative control. Each group contained 100–150 eggs. The injection concentration of the plasmid for the following EGFP mutation experiment was chosen based on the survival rate and positive rate of transgene.

Then, the linearized pGFP-RFP plasmid was injected at the chosen concentration, accompanied with TALENs mRNA, Cas9 mRNA/gRNA, or Cas9 nuclease/gRNA at gradient concentration, into the animal pole of one-cell stage embryos, with each group containing 100–150 embryos. To reduce the pre-reaction of Cas9 nuclease/gRNA with target plasmid, they were mixed just before injection. The concentration of the injection mixture for the following *ntl* mutation experiment was chosen based on the survival rate and mutation rate of target site.

The Cas9 nuclease/gRNA targeting *ntl* gene of sterlet was injected into 10 fertilized eggs at the chosen concentration.

### Identification of Gene Transferring

All injected embryos (*n* = 2456) were hatched at 16°C. The survival rates were calculated at neurula stage (2 days after fertilization, 2 dpf), transformed to arcsine square root and analyzed by ANOVA followed by Student–Newman–Keuls method. Differences were considered significant at *P* < 0.05.

**FIGURE 1:**
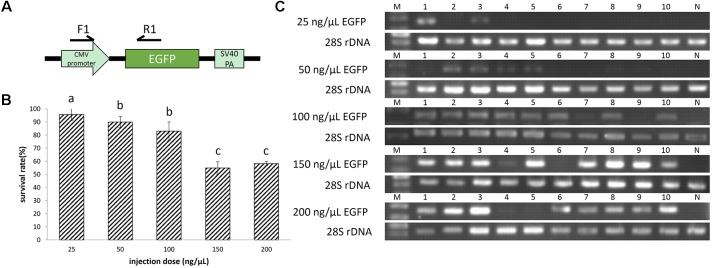
**(A)** The enhanced green fluorescent protein (EGFP) unit in the plasmid pEGFP-C1. **(B)** Survival rates of EGFP transgenic embryos. A total of 624 embryos were injected. The survival rate was transformed to arcsine square root, then analyzed by ANOVA followed by Student–Newman–Keuls method. Symbols with the same indicate groups that are not significantly different. **(C)** Detection of EGFP transgene by PCR. M represented marker, lane 1–10 represented 10 individuals, and N represented negative control.

All embryos (*n* = 624) injected with pEGFP-C1 plasmid were observed under a Leica M165FC stereoscope with a green fluorescence filter. At 2 dpf, 10 embryos were randomly sampled from each group injected with different plasmid concentrations. PCR was performed using the sampled genomic DNA. Part of the plasmid was amplified by a pair of primers F1 and R1 (**Table 1**), which spanned the CMV promoter and the EGFP coding sequence (**Figure 1A**). Part of 28S rDNA was amplified as an internal positive control by primers F2 and R2 (**Table 1**). The program for PCR was as follows: 95°C for 2 m, 30 cycles of 95°C for 30 s, 55°C for 15 s, and 72°C for 30 s, then 72°C for 5 m. The transfer rate of EGFP was estimated based on the number of positive PCR divided by the total number of embryos examined, as previously described (Li and Tsai, 2000).

**Table 1:** Primers sequence.

Primer	Sequence (from 5′ to 3′)
F1	TAGCGGTTTGACTCACGG
R1	TAGGTCAGGGTGGTCACGAG
F2	AAGTTGAAAAGAACTTTGAAGAGA
R2	GTTAGACTCCTTGGTCCGTGT
F3	GGTCGAGCTGGACGGCGACGTAAAC
R3	CCTCGGCGCGGGTCTTGTAGTTGCC
F4	GGAGAGCGAATTTCAGAA
R4	GCGCAATGTCATTTTAATAC

### Identification of Gene Editing

All embryos (*n* = 1832) injected with pGFP-RFP plasmid were observed with green and red fluorescence filters. At 2 dpf, 10 embryos were randomly collected from each group and genomic DNA was extracted. PCR was used to amplify the DNA region including the EGFP target site by primers F3 and R3 (**Table 1**), followed by a denature and then a slow cooling to 37°C. PCR products were separated on 8% polyacrylamide gels (PAGE) in 1× TBE buffer using the Mini-PROTEAN Tetra electrophoresis unit (Bio-Rad) at 100 V for 1.5 h. After staining with ethidium bromide, the gels were photographed and analyzed to calculate the mutation rate based on band intensity, as previously described (Chen et al., 2012). Mutation rates for each group were transformed to arcsine square root, then analyzed by ANOVA followed by Student–Newman–Keuls method. Differences were considered significant at *P* < 0.05.

The *ntl* gene was targeted in embryos and genomic DNA was extracted from each survived embryo at pre-hatching stage. The primers F4 and R4 (**Table 1**) were used to amplify the region including the first exon of the *ntl* gene. Mutation rates were analyzed by PAGE electrophoresis.

## Results

### Gene Transferring in Sterlet

When the pEGFP-C1 injection concentration increased from 25 to 200 ng/μL, the survival rate showed a declining trend, from 96 to 58%. And the survival rate at 100 ng/μL injection concentration is not significantly different from that at 50 ng/μL injection concentration (**Figure 1B**).

**FIGURE 2:**
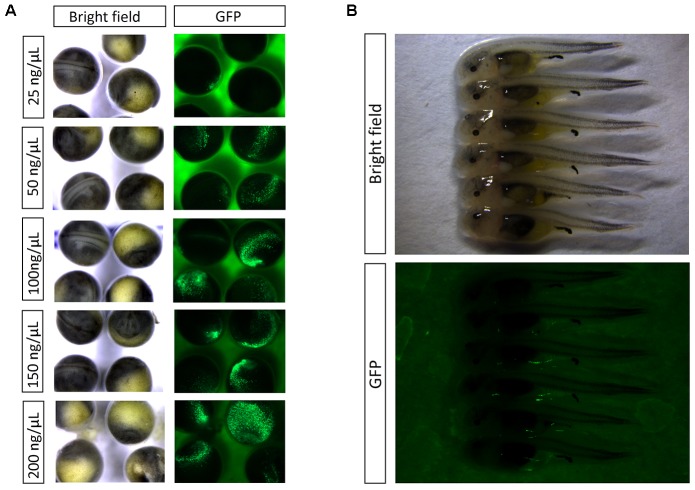
**(A)** Detection of EGFP by fluorescence in embryos at 2 dpf. **(B)** Detection of EGFP by fluorescence in embryos at 8 dpf.

Based on green fluorescence observed in embryos, the EGFP transfer rate increased with injected plasmid concentration. The transfer rate was nearly 20% when the injection concentration was 25 ng/μL. Using a concentration of 50 ng/μL, the transfer rate was approximately 40%, with weak fluorescence in embryos. When injected with 100 ng/μL plasmid, the fluorescence in embryos was enhanced, with an estimated 80% transfer rate. Finally, the transfer rate reached above 90% when injection concentration was 150 and 200 ng/μL, and the fluorescence was strong (**Figures 1C**, **2A**). At 8 dpf, there were dots of fluorescence in fry bodies, which represented the foreign EGFP DNA unit that had been integrated into the cell genome (**Figure 2B**).

Based on the result of pEGFP-C1 plasmid transfer, injection concentration of 100 ng/μL should be chosen for gene transfer, at which high survival rate (83%) and gene transfer rate (80%) could be achieved.

### Efficient Mutation Targeting Exogenous EGFP in Sterlet

100 ng/μL of linearized pGFP-RFP plasmid was injected into the animal pole of one-cell stage embryos, accompanied by TALENs mRNA, Cas9 mRNA/gRNA, or Cas9 nuclease/gRNA.

**FIGURE 3:**
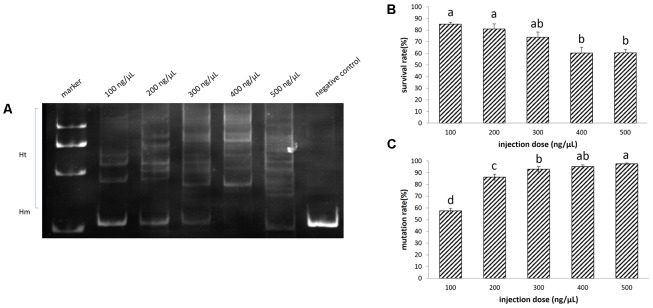
Disruption of EGFP by transcription activator-like effector nucleases (TALENs) in sterlet. A total of 532 embryos were injected. **(A)** Detection of mutation by PAGE. **(B)** Survival rate of injected embryos. **(C)** Mutation rate at target site. The survival rate and mutation rate were transformed to arcsine square root, then analyzed by ANOVA followed by Student–Newman–Keuls method. Symbols with the same indicate groups that are not significantly different.

Increasing the injection concentration of TALENs mRNA from 100 to 500 ng/μL, the survival rate declined from 85 to 60% (**Figure 3B**). Meanwhile, the mutation rate rose from 57 up to 98% (**Figures 3A,C**). The survival rate (74%) of embryos at neurula stage and the mutation rate (93%) reached a balance when the injection concentration was 300 ng/μL of TALEN mRNA. We sequenced 20 randomly selected clones containing DNA near the target site of EGFP, and identified 18 mutated sequences (Supplementary Figure 3).

**FIGURE 4:**
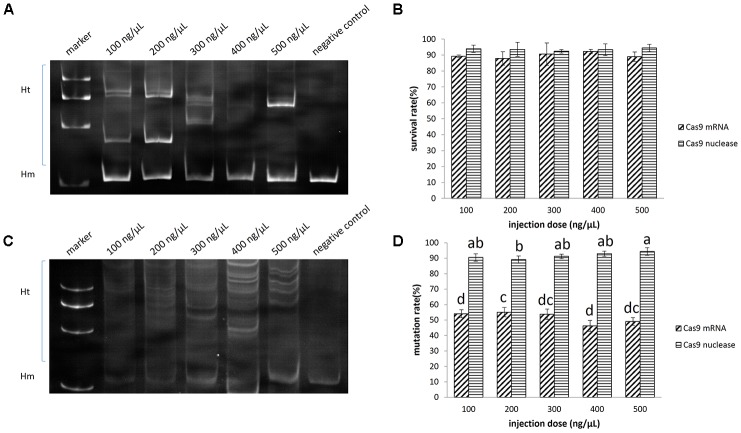
Disruption of EGFP by CRISPR/Cas9 in sterlet. A total of 682 embryos were injected with Cas9 mRNA, and 625 were injected with Cas9 nuclease. **(A)** Induced by Cas9 mRNA. **(B)** Induced by Cas9 nuclease. **(C)** Survival rate of injected embryos. **(D)** Mutation rate at target site. The survival rate and mutaion rate were transformed to arcsine square root, then analyzed by ANOVA followed by Student–Newman–Keuls method. Symbols with the same indicate groups that are not significantly different.

It appeared as though the concentration of the injected Cas9 mRNA was not related to the survival rate of embryos. The survival rate ranged between 88 and 92% when the injection concentration increased from 100 to 500 ng/μL (**Figure 4B**), while the mutation rate at the target site ranged between 46 and 55% (**Figures 4A,D**).

The mutation rate at the target site induced by the Cas9 nuclease ranged between 91 and 94%, which was significantly higher than that induced by Cas9 mRNA (*P* < 0.05) (**Figures 4C,D**). Since as low as 100 ng/μL Cas9 nuclease could lead to 91% mutation (no significant difference from other groups) and 94% embryo survival, we chose this concentration for *ntl* mutation experiment. We further identified 19 clones containing mutated DNA out of 20 by sequencing (Supplementary Figure 4).

**FIGURE 5:**
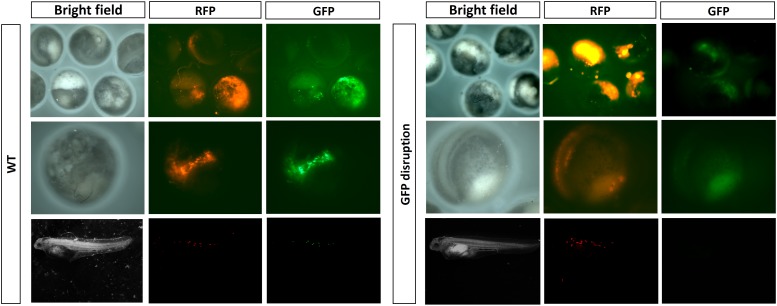
Detection of EGFP disruption by fluorescence.

Under a stereoscope, we observed the reduction or even complete disappearance of green fluorescence in the embryos and fries which had a high mutation rate of EGFP (**Figure 5**).

### Efficient Mutation Targeting Endogenous *ntl* Gene in Sterlet

**FIGURE 6:**
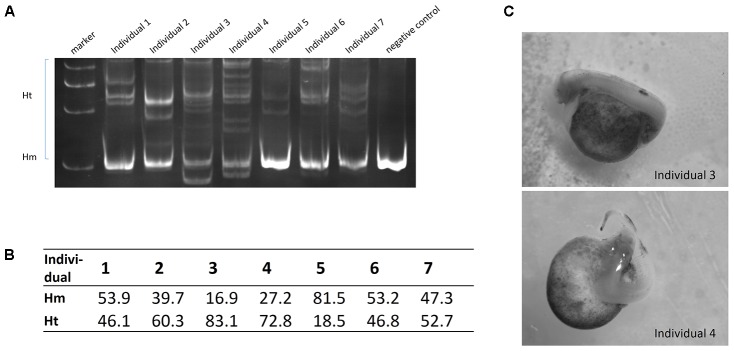
**(A)** Detection of *ntl* gene mutation by PAGE. **(B)** Mutation rate (Ht%) of individuals. **(C)** Phenotype of *ntl* disruption.

The *ntl* gene was chosen as an endogenous target to test the validity of Cas9 nuclease in sterlet. Three gRNAs were each mixed with 100 ng/μL Cas9 nuclease before injection. We detected obvious target site mutations in genomic DNA mixtures from one injected group, and randomly selected seven fries from this group to measure mutation rates in individuals. The mutation rate ranged from 18 to 83% (**Figure 6A**). The two individuals with the highest rate (73 and 83%) showed visible phenotype of bent spine and twisted tail (**Figures 6B,C**). The genome DNA was extracted from the fry with 83% mutation rate, and 10 randomly selected clones containing DNA near the target site of *ntl* were sequenced, in which 8 mutated sequences were identified (Supplementary Figure 5A).

## Discussion

In this study, gene transfer and gene editing technologies were established in sterlet. We repeated the micro-injection experiments several times. Although the embryo survival rate varied due to the quality of different batches of eggs, the gene transfer rate and mutation rate were kept in a stable range, which proves the reliability and repeatability of these technologies in sterlet. Both TALENs and Cas9 nuclease could efficiently mutate the EGFP reporter gene. Moreover, Cas9 nuclease induced high mutation rates in an endogenous gene, with low toxicity to embryos. This suggests that the CRISPR/Cas9 nuclease technology is a better choice for gene editing than TALENs in sterlet.

There are physical, chemical and biological methods to achieve gene transfer in fish, including microinjection, sperm mediated gene transfer (SMGT), gene gun, electroporation, virus mediated gene transfer, etc. Microinjection is extensively used owing to its low cost, ease of visualization, and high efficiency (Tonelli et al., 2017). Generally speaking, microinjection is only applicable for those eggs having clear animal pole with soft and transparent envelope (Hostetler et al., 2003). From this view, microinjection is not suitable in sturgeon, due to the black non-transparent eggs covered with rigid envelope (Sin et al., 1997). Thus, we preferred to try two other methods commonly used in fish—SMGT and gene gun. SMGT can introduce foreign genes directly into the cell nuclear regardless of the characteristics of eggs, which was successfully applied in more than 20 fish species such as zebrafish and silver sea bream (*Sparus sarba*) (Khoo, 2000; Lu et al., 2002). However, in sterlet, we obtained gene transferring rate as low as 1/534 (Supplementary Figure 6). Gene transfer using gene gun is a simple, high-throughout method, which was successfully practiced in zebrafish and rainbow trout (Zelenin et al., 1991; Lee et al., 2000). However, in sterlet, we obtained only one egg expressing green fluorescent, out of 268 survived embryos (Supplementary Figure 7). Finally, we attempted microinjection in sterlet and found that within 20 min after fertilization, the micropyle was clear and the envelope was soft enough for a capillary needle (with enough rigidness) to penetrate (Supplementary Figure 8). This finding made microinjection and gene transferring possible. Moreover, we tried Tol2 transposase, which was originally from medaka and could remarkably increase integration rates of foreign genes in zebrafish and *Xenopus tropicalis* (Kawakami et al., 2000; Hamlet et al., 2006), in sterlet transgenesis. However, the sterlet embryos injected with mixture of Tol2 transposase mRNA and the plasmid containing CMV-EGFP-polyA fragment flanked with Tol2 recognition sites didn’t show stronger fluorescence, compared with the embryos injected with the single plasmid (Supplementary Figure 1). More tools for effective integration in sturgeons need to be digged out in future study.

Transcription activator-like effector nucleases and CRISPR/cas9 have been tested in a variety of organisms and showed extensive applicability (Ma and Liu, 2015; Gaj et al., 2016; Sovová et al., 2017). Since the efficiency for endonuclease to bind and cut genomic DNA was not only affected by the sequence of the target site, but also by the chromatin structure near the target site, we first tested the possibility of gene editing using an exogenous DNA fragment, EGFP, as a target. Prior to this, we confirmed the validity of chosen target sites in zebrafish. Injection of TALENs mRNA or Cas9 mRNA/gRNA at 200 ng/μL could induce 62 or 68% mutation in the EGFP sequence in zebrafish, respectively (Supplementary Figure 9). Subsequently, we tested the same system in sterlet.

The cutting efficiency of TALENs was improved mainly by modification of FokI subunit (Doyon et al., 2011). The TALENs used in this study, with optimized in FokI structure, could produce mutation rates of more than 50% in zebrafish and *Xenopus tropicalis* (Liu et al., 2014). While in sterlet, the same TALENs system could induce EGFP mutation as high as above 90% and keep 70% of the embryos alive. Beside the easier binding between the nuclease protein and the fragmented EGFP site, the more important reason for the high cutting efficiency might be the slow development of sturgeon embryos (first cleavage happens at 3 h after fertilization in 16°C), during which the endonuclease may have worked for a longer time at one and two-cell stages. Similarly, efficient gene editing was also achieved in the founder fish of tilapia, common carp and rice field eel, whose embryos all developed slowly (Li et al., 2014; Zhong et al., 2016; Feng et al., 2017).

The cutting efficiency of CRISPR/Cas9 is directly related to the translation of Cas9 nuclease protein, which is the reason why codon optimization can improve the cutting efficiency (Cong et al., 2013). In research on fish, there is only codon optimized Cas9 mRNA for zebrafish, which has been shown to induce a 75–99% mutation rate of endogenous genes in zebrafish (Jao et al., 2013). However, the same Cas9 mRNA induced no more than a 55% mutation rate in sterlet, probably due to the distant evolutionary relationship between zebrafish and sterlet which reduced the efficiency of Cas9 mRNA translation. Instead, when we directly transferred Cas9 nuclease protein at 100 ng/μL, both the mutation rate and embryo survival rate increased above 90%. In contrast with TALENs, CRISPR/cas9 gave better survival rate. We therefore recommended the use of Cas9 nuclease/gRNA to target genes in sterlet.

Furthermore, we successfully mutated an endogenous gene, *ntl*, and obtained sterlet fries with phenotypes similar to those observed after *ntl* knockdown using morpholino technology in zebrafish (Amack and Yost, 2004). Moreover, we edited another endogenous gene, *dickkopf1*, with Cas9 nuclease and mixture of three designed gRNA, and successfully induced mutation in *dickkopf1* gene (Supplementary Figure 5B). Most reports on gene editing in polyploid species are based on plants research such as rice (*Oryza sativa*), sugarcane (*Saccharum* spp. *hybrids*), *Camelina sativa* and *Arabidopsis thaliana* (Shan et al., 2013; Jung and Altpeter, 2016; Aznar-Moreno and Durrett, 2017; Ryder et al., 2017). *tyr* and *pax6* were mutated in tetraploid *Xenopus tropicalis* using TALENs and embryos with loss of pigment or malformed eyes were obtained (Suzuki et al., 2013). In this study, efficient mutation of endogenous gene in tetraploid sterlet suggested applicability of Cas9 nuclease to be applied in other sturgeon species with hexaploid or octoploid genomes.

## Author Contributions

WH and HH: conceived and designed the experiments. JC, WW, ZT, YD, and TD: performed the experiments. JC and WW: analyzed the data and interpreted the results. HH, HZ, and ZZ: contributed reagents/materials/analysis tools. JC, WH, and HH: wrote and revised the paper.

## Conflict of Interest Statement

The authors declare that the research was conducted in the absence of any commercial or financial relationships that could be construed as a potential conflict of interest.
