# Supplementary material for: Efficient Gene Transfer and Gene Editing in Sterlet (Acipenser ruthenus)
Source: Front Genet. 2018 Apr 6;9:117. doi: 10.3389/fgene.2018.00117 (PMC5897424; doi:10.3389/fgene.2018.00117)
Supplement: Supplementary file 1 [file Data_Sheet_1.docx]

Electronic supplementary material


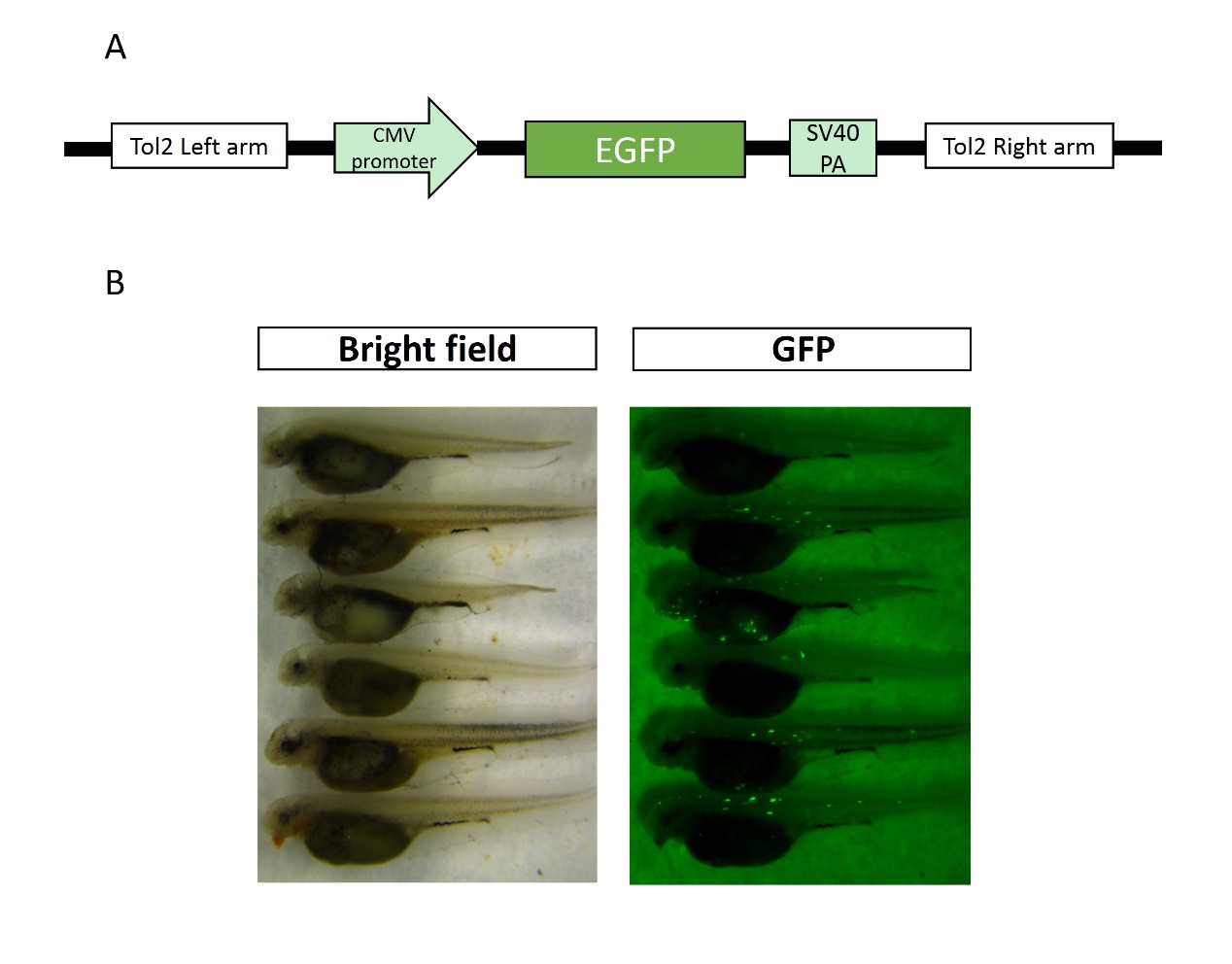


Supplementary figure 1. Assessment of Tol2 transposase effect in sterlet by fluorescence. A, The Tol2 recognition sites were inserted into the plasmid pEGFP-C1, flanking CMV-EGFP-polyA fragment, to construct pEGFP-Tol2. Tol2 transposase mRNA was obtained by *in vitro* transcription from the plasmid T7-Tpase **(Addgene plasmid 51818).** Then the mRNA and pEGFP-Tol2 were injected together with a concentration ratio of 2:1. B, From up to bottom were embryos injected with 50 **ng/μL linearized** pEGFP-C1, 100 **ng/μL linearized** pEGFP-C1, 150 **ng/μL linearized** pEGFP-C1, 50 **ng/μL** pEGFP-Tol2 with 100 **ng/μL** transposase mRNA, 100 **ng/μL** pEGFP-Tol2 with 200 **ng/μL** transposase mRNA, and 150 **ng/μL** pEGFP-Tol2 with 300 **ng/μL** transposase mRNA, orderly.


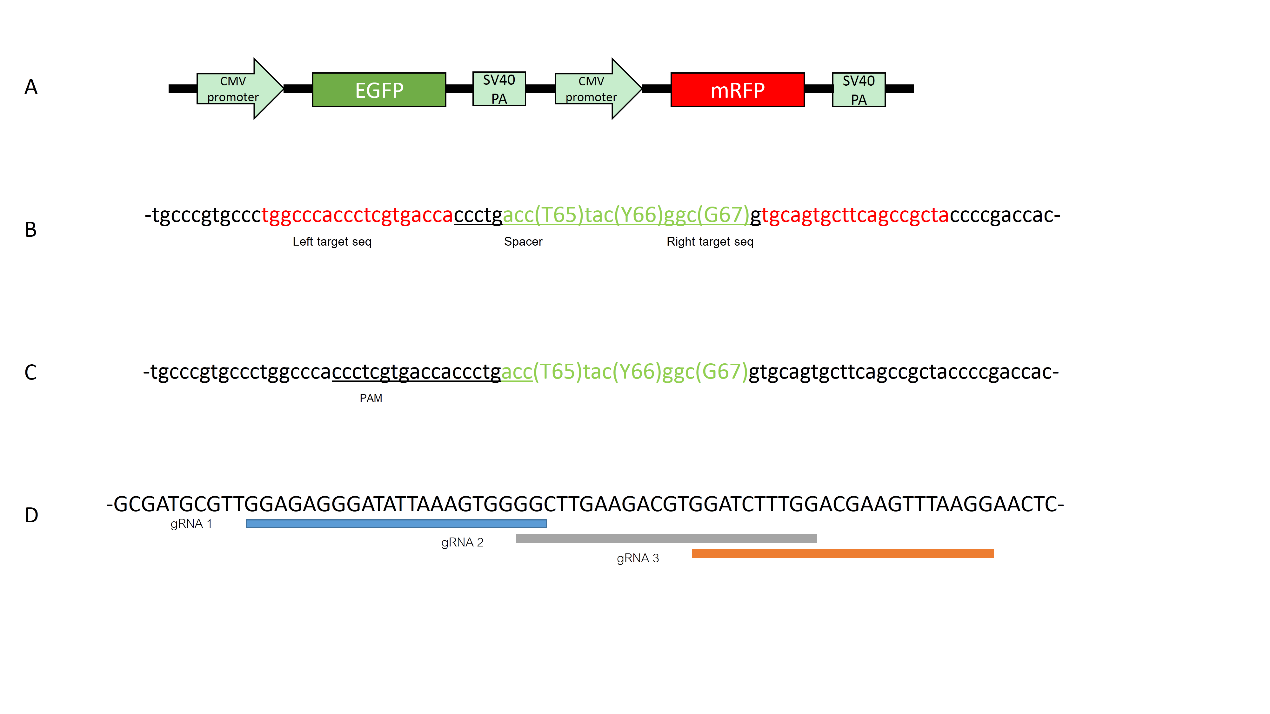


Supplementary figure 2. A, Construction of the plasmid pSKD-RFP. B, Design of EGFP TALENs target site. C, Design of EGFP CRISPR/Cas9 target site. D, Design Cas9 target sites on *ntl* gene.


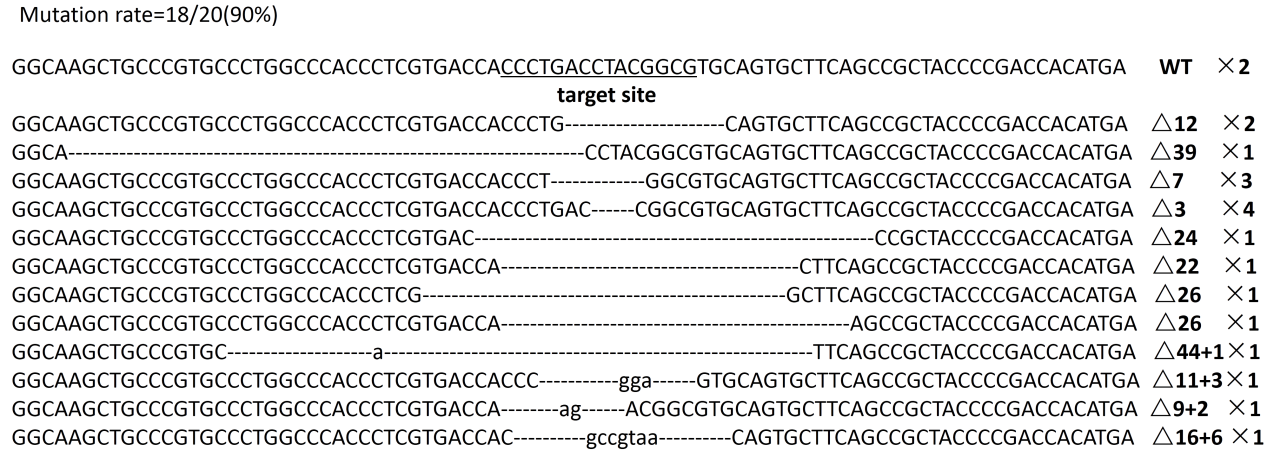


Supplementary figure 3. Genotypes of EGFP induced by 300 ng/μL TALENs.


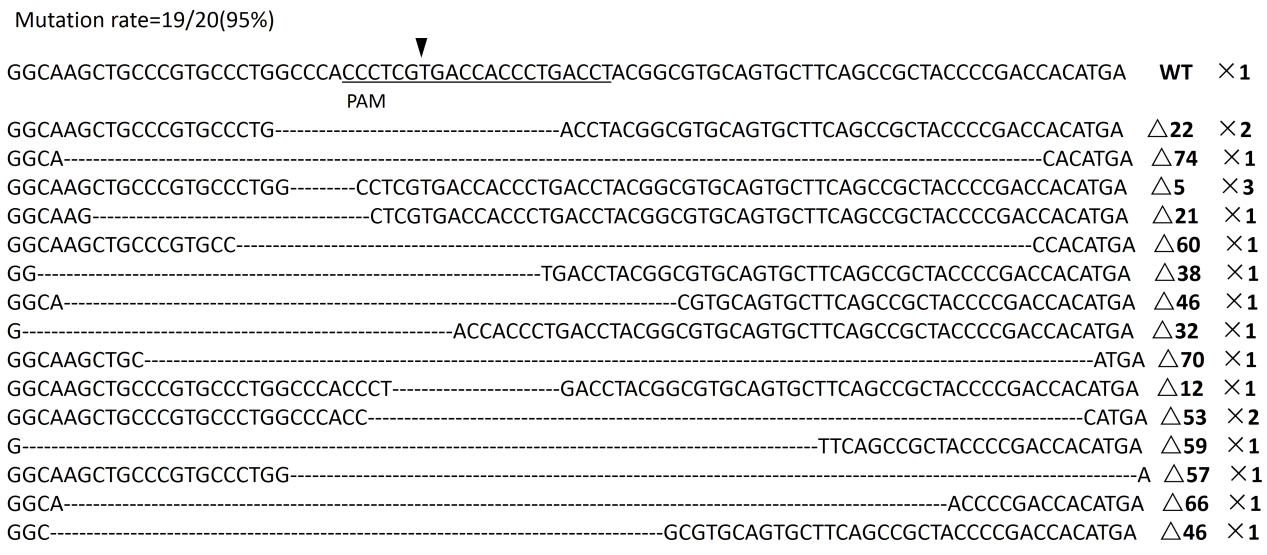


Supplementary figure 4. Genotypes of EGFP induced by 100 ng/μL Cas9 nuclease. ▼indicates the site from which the genomic DNA was cut off.


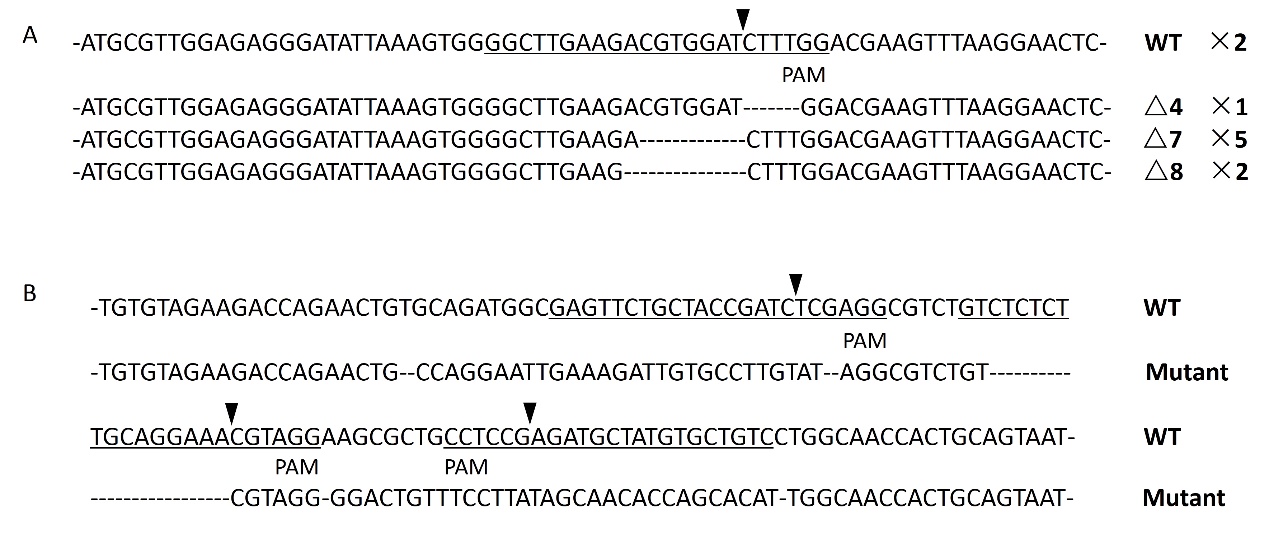


Supplementary figure 5. A, Genotypes of *ntl* mutant induced by Cas9 nuclease. B, Genotype of *dickkopf1* mutant induced by Cas9 nuclease mixed with three gRNA. ▼indicates the site from which the genomic DNA was cut off.


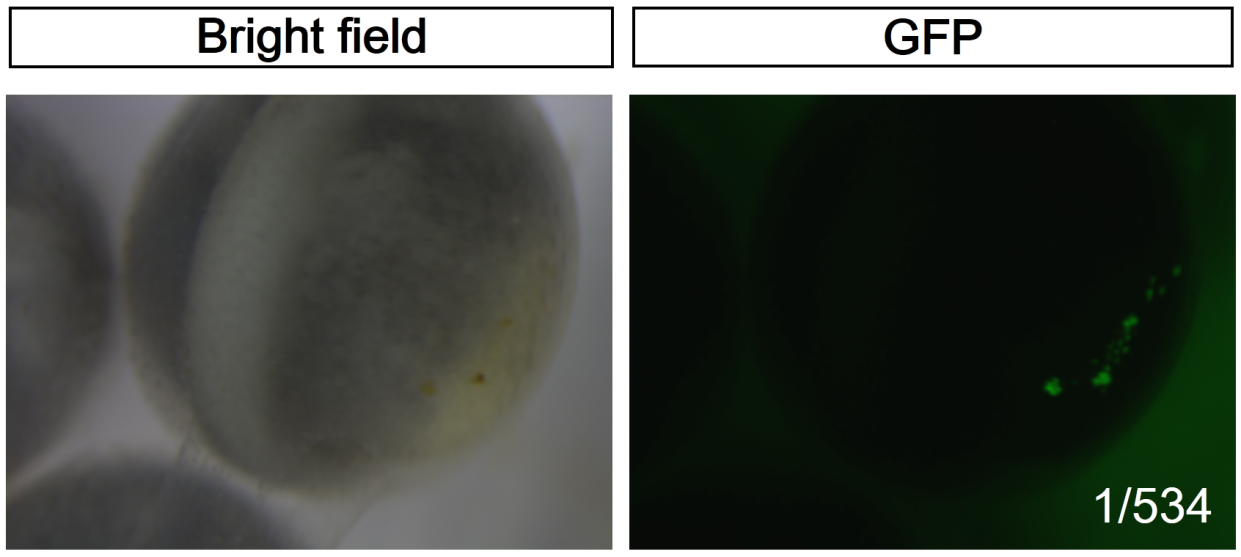


Supplementary figure 6. Sperm mediated EGFP transfer. The semen was dropped into the electroporation cuvettes and eletroporated under 1400V, 1600V, 1800V, 2000V, 2200V or 2400V. The maximum tolerated voltage (2000V) was determined when about 60% of sperm remained vital. Then the mixture of semen and the pEGFP-C1 plasmid was eletroporated using 2000V, followed by an immediate fertilization. At 2dpf, a total 534 embryos survived and green fluorescence was observed in only one embryo.


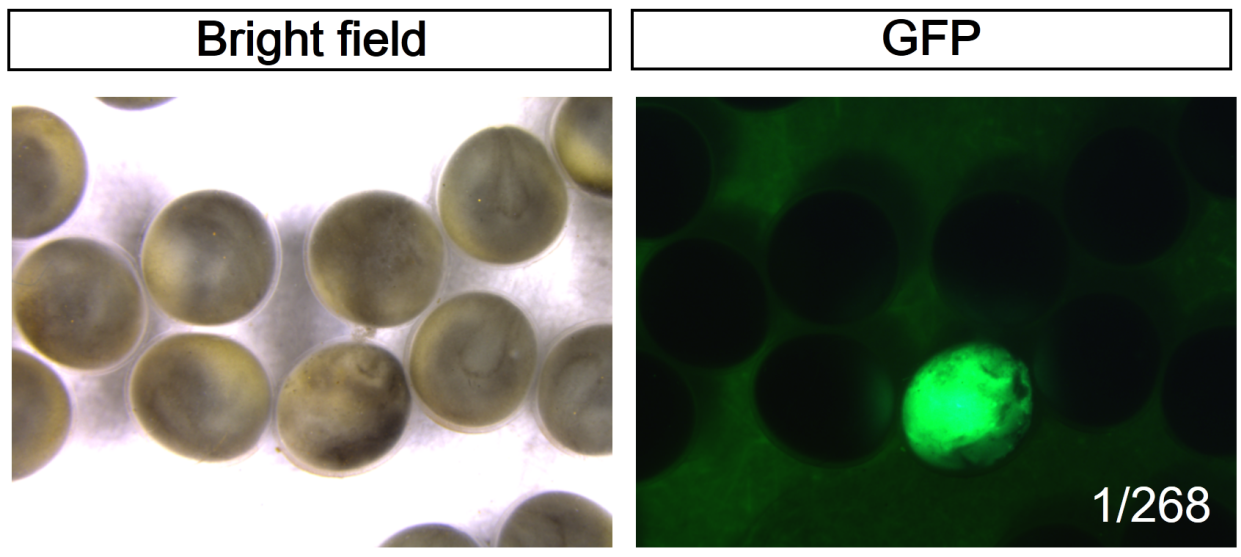


Supplementary figure 7. EGFP transfer by gene gun. 50 μg pEGFP-C1 plasmid was packaged by gold particle, and bombed into fertilized eggs using a Bio-Rad PDS-1000/He gene gun, under a vacuum pressure of 1350psi and distance of 6 centimeter. At 2dpf, a total 268 embryos survived and green fluorescence was observed in only one embryo.


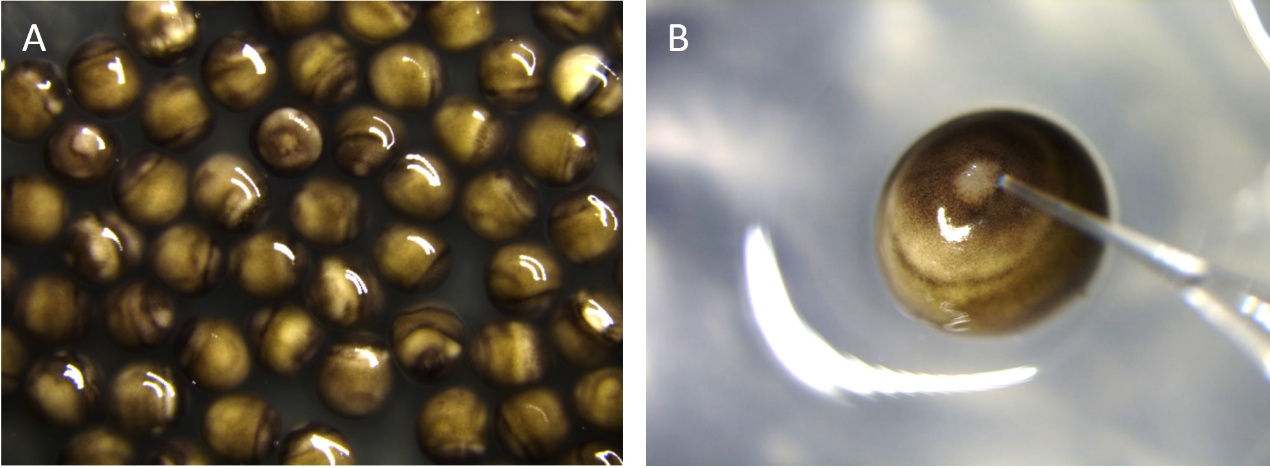


Supplementary figure 8. A, Fertilized eggs of **sterlet**. B, Microinjection by a glass capillary needle.


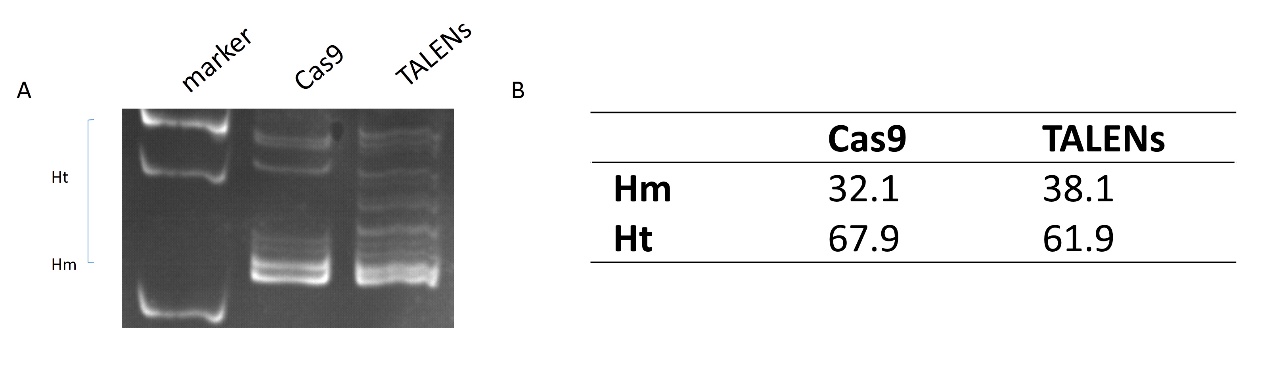


Supplementary figure 9. Disruption of EGFP in zebrafish. A, Detection of EGFP mutation by PAGE. B, EGFP gene mutation rate (Ht%).
